# The role of economic evaluations in advancing HIV multipurpose prevention technologies in early-stage development

**DOI:** 10.3389/frph.2024.1272950

**Published:** 2024-04-18

**Authors:** Katerina Chapman, Sergio Torres-Rueda, Mutsumi Metzler, Bethany Young Holt, Elijah Kahn-Woods, Douglas Thornton, Gabriela B. Gomez

**Affiliations:** ^1^ Global Access, IAVI, New York, NY, United States; ^2^ Department of Global Health and Development, London School of Hygiene and Tropical Medicine, London, United Kingdom; ^3^ Medical Devices and Health Technologies, PATH, Seattle, WA, United States; ^4^ CAMI Health, Initiative for MPTs, Public Health Institute, Sacramento, CA, United States; ^5^ Global Health Training, Advisory and Support Contract, United States Agency for International Development, Washington, DC, United States

**Keywords:** economic evaluation, low- and middle-income countries, multipurpose prevention technologies, HIV, prevention, product development, research and development

## Abstract

Product development is a high-risk undertaking, especially so when investments are prioritized for low- and middle-income countries (LMICs) where markets may be smaller, fragile, and resource-constrained. New HIV prevention technologies, such as the dapivirine vaginal ring (DVR) and long-acting injectable cabotegravir (CAB-LA), are being introduced to these markets with one indication, meeting different needs of groups such as adolescent girls and young women (AGYW) and female sex workers (FSWs) in settings with high HIV burden. However, limited supply and demand have made their uptake a challenge. Economic evaluations conducted before Phase III trials can help optimize the potential public health value proposition of products in early-stage research and development (R&D), targeting investments in the development pathway that result in products likely to be available and taken up. Public investors in the HIV prevention pipeline, in particular those focused on innovative presentations such as multipurpose prevention technologies (MPTs), can leverage early economic evaluations to understand the intrinsic uncertainty in market characterization. In this perspective piece, we reflect on the role of economic evaluations in early product development and on methodological considerations that are central to these analyses. We also discuss methods, in quantitative and qualitative research that can be deployed in early economic evaluations to address uncertainty, with examples applied to the development of future technologies for HIV prevention and MPTs.

## Introduction

Economic evaluations analyze new products or technologies in comparison with already available ones to assess incremental cost and health impact (1). They collate the available evidence up through the current stage of development and can use modelling, addressing uncertainty linked to incomplete trial data, variable clinical pathways, future costs, broadly defined markets, among others (2). These economic evaluations are usually conducted in late-stage product development (i.e., once safety and initial data on efficacy have been collected), to inform introduction and reimbursement decisions. However, the information gained when economic evaluations are conducted early in the research and development (R&D) process allows funders, future investors, and product developers to prioritize resources and support resource allocation decisions across portfolios. Early insights are of relevance when investing in products prioritized for access in low-and-middle income countries (LMICs). LMIC markets experience unique challenges such as overburdened health care systems, new and complex regulatory systems, and limited resources or multiple payers with different decision making criteria, contributing to a less predictable market (3).

Currently, HIV infection remains a global challenge, with approximately 1.3 million [1.0 million–1.7 million] new infections in 2022 (4), 51% of which were reported in sub-Saharan Africa (SSA) (4). Although substantial decreases in HIV transmission and AIDS-related deaths were observed between 2005 and 2015, mainly due to the scale up of antiretroviral therapy (ART) for treatment and for prevention, the rate in reduction of new infections has plateaued in recent years (4). Of an estimated 250,000 [150,000–360,000] adolescent girls and young women (AGYW) acquiring HIV in 2021 globally, 82% of them were living in SSA (5), with new HIV infections among AGYW declining slower than among their male counterparts (rate of decline 42% vs. 56%, respectively) (5). Reducing HIV transmission among key populations, such as female sex workers (FSWs), AGYW and pregnant and breastfeeding people (PBFP), remains an important challenge, particularly in SSA (5).

An expanding number of biomedical HIV prevention technologies with demonstrated efficacy in clinical trials and, where relevant, feasibility and acceptability data are either currently available or soon to be available (6). These include daily oral antiretroviral (ARV) pills for pre-exposure prophylaxis (PrEP), the monthly dapivirine vaginal ring (DVR) and long-acting cabotegravir for HIV prevention (CAB-LA), an intramuscular injectable form of PrEP (7). Efficacy studies in SSA have shown monthly DVR and bi-monthly CAB-LA to effectively reduce the risk of HIV infection. Monthly DVR demonstrated a reduction in the risk of HIV infection among African women of 31% and 27% in two Phase 3 multi-site placebo-controlled studies (8, 9) and HPTN 084 reported the risk of HIV infection in the injectable cabotegravir group was reduced by 91% compared to the control group using oral PrEP (10). There are data to suggest implementation of the monthly DVR is feasible, while acceptability data are mixed. Some studies show the ring to be more acceptable than oral PrEP to AGYW while other studies suggest that acceptability varies across countries and usage during sex and menses (11). Data from the DELIVER and B-PROTECTED studies suggest DVR is safe to use during pregnancy and breastfeeding (12). However, the introduction and uptake of these products has been limited due to supply and demand challenges. DVR was prequalified and was recommended by the World Health Organization in 2020 and 2021, respectively, and regulatory approval from several countries in SSA has followed since then (13). Yet, it was not added to the South African Essential Medicines List (14), which guides the national health agenda, due to lack of studies comparing it to the current standard of care of oral PrEP and it being considered expensive at the initially proposed price of R300 per month compared to R52 for oral PrEP (14). Additionally, while CAB-LA has shown to be safe and efficacious and early implementation studies suggest high adherence rates (15), its introduction has been limited due to challenges relating to supply barriers in LMICs, implementation hurdles, and price (16). In 2022, ViiV, the product developer, signed a voluntary license with Medicines Patent Pool to enable manufacture of CAB-LA by generic companies, aiming to improve availability medium term (17).

While these efforts are underway to improve the introduction and scale up of currently or soon-to-be available HIV biomedical interventions, further work is needed to ensure future HIV prevention options meet women's varied needs including expanding choice by diversifying HIV prevention offering. Ongoing early development focuses on innovative combinations such as multipurpose technologies (MPTs), which aim to address the multiple needs of AGYW and others who are at risk for HIV, other STIs, and unwanted pregnancies (18). There are currently a number of MPTs in the pipeline such as oral pills, long acting injectables and implants with dual indication for HIV prevention and the prevention of unwanted pregnancies (19). Despite this agenda, investment for new HIV prevention products has flattened over the last eight years (20, 21). Continued engagement including identification of commercial partners as these novel MPTs move through clinical development is needed. MPTs represent a unique business case, providing a potential dual market in both high-income countries (HIC) and LMICs.

Economic evaluations have become common in preparation for market introduction as part of health technology assessments (HTAs) with the intention of establishing cost-effectiveness for payer coverage (2). Opportunities to shape a product's target profile, business case and its readiness for introduction can be created by undertaking economic evaluations earlier in the development pathway.

**Figure 1:**
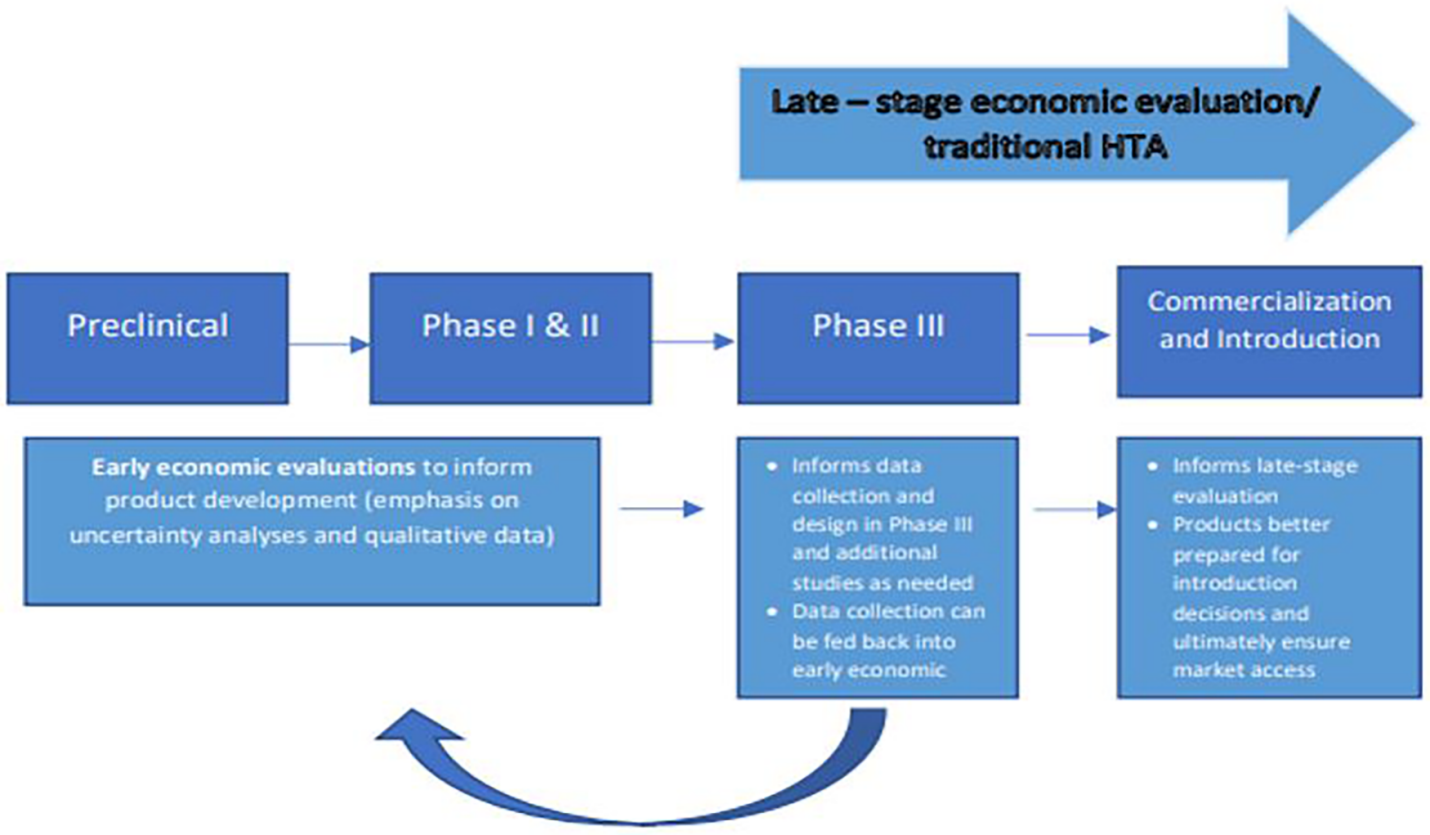
Illustration of product development process and example of the value early economic evaluation can provide if integrated into the development pathway, leading to products better prepared for introduction.

Undertaking economic evaluations earlier in development poses a few challenges. The treatment of uncertainty is one of them. Therefore, an economic evaluation conducted early in development often relies on both quantitative and qualitative methods to address this uncertainty in the absence of observed estimates. Because economic evaluations at this stage are frequently conducted in-house, there are limited examples available to the public and limited methodological guidance. This can result in omissions of costs, inappropriate comparators and characterization of uncertainty or assumptions, among others (22). However, there are use case examples for how these evaluations are leveraged for internal decision making (2, 23) and to mitigate the risks (23) and high costs of late-stage development (23). In this context, they can provide valuable insights into clinical trial design (23, 24), into target populations or other drivers that improve value for money (25, 26) and can guide decisions on what data need to be collected at the next phase of development (27) so that uncertainty is reduced when introduction decisions are made by policy makers and payers. Importantly, these early analyses can inform product developers' decisions on how to improve a product's eventual value for money, helping refine the target product profile as well as informing which product to prioritise across a given portfolio, focusing resources on those most promising products for further development and those most appropriate for a future programme (Figure 1).

Here we reflect on two methodological considerations that are central to early economic evaluations (approaches to uncertainty and the role of qualitative research). Either qualitative or quantitative data can be used to inform economic evaluations. In particular, early in the product development, researchers will focus on literature reviews and expert elicitation to parameterize model-based economic evaluations. We have described two methodological aspects of model-based economic evaluations that are key at this stage, namely, the use of qualitative data to inform model parameters and structure and the value of uncertainty analyses as main outputs to inform decisions. Using examples, we provide snapshots on how they might be applied to the context of the HIV MPT development landscape. The purpose of this paper is to illustrate the mixed method approach needed in early R&D and its applicability to HIV MPTs. Although early economic evaluation examples are limited, these examples can be applied to the development of future technologies for HIV prevention and MPTs.

### Approaches to dealing with uncertainty

During early R&D, the data available to characterize products are limited and will evolve as the technology progresses in development. Data limitations include no efficacy, safety data, a limited target product profile, and limited awareness of the implementation pathway and usability of the product. To address these limitations, more attention has been paid in the last decade to the use of combined models of disease progression and pharmacodynamics linked to economic models (2, 22–24, 28–32). These linked models may then be used to inform trial design, guide strategic development decisions (33), and define and refine the target product profile and assumptions providing measurable value propositions, which emphasizes the need, the benefits, and its comparison to other products (34). Linked model outputs include estimates of efficacy, dosing regimen, pharmacokinetics, among others. These outputs can guide clinical development and help reframe value propositions once new data are collected during the different stages of clinical trials, providing an iterative framework for decision making (35, 36), future trial design (25), and the preparation of strategies for reimbursement and pricing (2, 29, 32, 37–39). Though researchers often emphasize the uncertainty that comes with modelling early in the development process as a limitation of early economic modelling (2, 22–24, 28–32), the framing and communication of this uncertainty becomes the objective of these analyses and future evidence generation will revolve around addressing this uncertainty. The framing of uncertainty and how to address it influences the methods used and the choice of output parameters. Deterministic and probabilistic sensitivity analyses as well as threshold analyses can be conducted across a broad range of parameters (e.g., expected efficacy with price, probable implementation strategies). These combinations help determine the viability of a future technology. Though similar analyses may be used later in product development, early insights that clarify trade-offs between product attributes inform developers on the specific product characteristics that could and must be optimised in future development.

For example, in the context of novel products for HIV prevention, Dugdale et al. selected three countries with a range of HIV epidemic characteristics to model cost effectiveness of novel HIV broadly neutralizing antibodies (bnAbs) for infant prophylaxis (25). Alongside a base case, bnAbs were modelled using sensitivity analysis across a range of varying parameters (e.g., efficacy, cost, different implementation strategies, different target populations) to determine parameter space of likely market feasibility (25). This information is critical, providing key parameters for product development targets for characteristics such as, efficacy, price, effect duration, and validating the potential cost effectiveness of implementation strategies to guide future HIV bnAb trials. As HIV prevention products and MPTs progress in development, evaluating the drivers of cost effectiveness can be used to tailor trials, guide future data collection, and better prepare a product for introduction (through complete evidence packages), increasing the likelihood of affordable, acceptable, scalable, and widely available technologies.

### Central role of qualitative data

The context and setting where the future technologies will be delivered is one of the key determinants of market viability and return on investment. Expert elicitation is key to synthesize opinions of stakeholders and fully understand the use case and context of a potential technology. Unlike in later-stage economic evaluations where data is more readily available, early economic evaluations place a heavier emphasis on expert opinions (2, 29, 32, 37–39) that can be used to complement the literature review, the existing trial data, and to illustrate uncertainty (29) in the absence of empirical data. For example, focus groups and key informant interviews (KIIs) can be used to validate assumptions and models (40). These expert opinions address uncertainty in the clinical pathway (32) through identification of clinical endpoints or patient target groups for testing as well as delivery strategies that may be possible in the future and their challenges (32). However, methods to conduct expert elicitation and to analyse the qualitative results are not standardized (41). Despite the lack of consensus on methods, expert opinion can help anchor key assumptions in early analyses (41). In the absence of performance data for an early-stage product, expert elicitation can also identify correlates that will serve as predictors of future performance. In the HIV prevention context, experts may use reference products such as oral, vaginal or injectable PrEP (6). An example is Unitaid's work when conducting conducted KIIs with global experts in HIV prevention, contraception and STIs, identifying key considerations for MPT development. Considerations included challenges to development and approval, but also variables and definitions guiding the development of an investment case, and the definition of decision points to advancing from pre-clinical to later stages of development (19). Key considerations identified by these KIIs can inform the MPT landscape for developers, providing insight into opportunities and challenges early in the development process. As public investors prioritize products for investment at an early stage of development, stakeholder inputs can help compensate for gaps in evidence, recommend implementation scenarios, and identify priority populations.

## Discussion

As useful as early economic evaluations are, they do present certain limitations. Using data from early trials may not reflect future clinical results or the ultimate patient population, making market viability difficult to assess. Additionally, it may not be possible to cover all possible scenarios and deciding the most important parameters to be considered will be essential yet mainly driven by the selection of stakeholders consulted. Data on future market competitors, public policy evolution, and manufacturing costs at scale will need to be estimated and arranged into scenarios where the likelihood of occurrence is unknown. However, in a time of growing development costs and with a higher proportion of funding for HIV prevention coming from public investment, MPTs can offer a unique business case, one with an expanded market and opportunities in both LMICs and HICs, making their development and commercialization feasible. Feeding into this business case, early economic evaluations provide an early look at implementation costs of a product, within target populations, and among indications that may improve PD efficiency and offer early insight into potential returns and economic feasibility. Additionally, investing resources into early and iterative economic modeling can produce stronger, better prepared products, and avoid the risk of expending resources carrying products through development that may be ill-suited for markets of interest.

Addressing uncertainty as one of the outputs of these early analyses can help improve upon decisions, model parameters, trial and product design, pricing, and lay groundwork for eventual market access. While stakeholder elicitation represents a resource to address evidence gaps, as data becomes available, these economic models can be further refined and improved in an iterative process. Finally, as products for HIV prevention and multipurpose prevention progress in development to phase 2 and 3, transparent business cases will facilitate engagement with commercial partners. Leveraging uncertainty analyses and qualitative data collection methods early on can refine the value proposition and strengthen those business cases, setting up products early for success.

## Data Availability

The original contributions presented in the study are included in the article, further inquiries can be directed to the corresponding author.
